# The Invisible Drivers of Dementia Disparities: A Systematic Review of SDOH in Alzheimer’s Patients and Caregivers

**DOI:** 10.1002/alz70861_108020

**Published:** 2025-12-23

**Authors:** Geovanni Vazquez, Lisa Carbonell

**Affiliations:** ^1^ Boston Medical Center, Springfield, MA USA; ^2^ Alzheimer Association, Springfield, MA USA

## Abstract

**Background:**

Social determinants of health (SDOH) play a critical role in shaping outcomes for individuals with Alzheimer’s disease and related dementias (ADRD) and their caregivers, yet their systemic impact remains understudied. This review examines how socioeconomic status, race/ethnicity, healthcare access, and social support influence disease progression, care quality, and caregiver burden.

**Method:**

A systematic search was conducted across PubMed, PsycINFO, CINAHL, and Scopus for peer‐reviewed studies (2000–2023) addressing SDOH in AD/ADRD patients and caregivers. Included studies were screened for relevance, with data extracted on key themes and disparities.

**Result:**

Findings reveal that lower income, education, and marginalized identities correlate with delayed diagnosis, accelerated cognitive decline, and reduced access to care. Caregivers facing financial strain or limited social support reported higher stress and worse mental health. Racial/ethnic minorities experienced compounded barriers, including cultural incompetence in healthcare systems.

**Conclusion:**

SDOH significantly exacerbate disparities in ADRD outcomes and caregiving experiences. The development of policies addressing structural inequities, such as extending Medicaid coverage and tailoring interventions based on cultural characteristics, is urgently required. Future research should investigate intersectional impacts and longitudinal effects of SDOH on dementia trajectories.

